# Supplementary material for: A mechanistic model of the BLADE platform predicts performance characteristics of 256 different synthetic DNA recombination circuits
Source: PLoS Comput Biol. 2020 Dec 18;16(12):e1007849. doi: 10.1371/journal.pcbi.1007849 (PMC7781486; doi:10.1371/journal.pcbi.1007849)
Supplement: S1 Fig — Schematic diagram of the tyrosine recombinase-mediated 2-input BLADE platform. Tyrosine recombinases Cre (red circles) and Flp (blue circles) are expressed constitutively at the same rate, α. LoxP sites are depicted as red triangles with different shades used to illustrate the result of recombination events. FRT sites are depicted as blue triangles with different shades used to illustrate the result of recombination events. White boxes depict each of the four BLADE addresses. Black arrows depict DNA:protein binding reactions comprising stable excision events. The rate of degradation of recombinase protein is denoted by βp. The rate of a Cre/Flp monomer binding reversibly to free loxP/FRT sites is denoted by k1, k-1; due to the cooperativity of monomer binding, we denote the rate of a Cre/Flp monomer binding reversibly to an occupied loxP/FRT site by k2, k-2. The rate of Holliday junction formation is denoted by k3, k-3. Each of the five Holliday junction strand exchanges are denoted by k4, k-4, k5, k-5, k6, k-6, k7, k-7, respectively. The rate of dilution of excised DNA due to cell division is denoted by δ. The empty set symbol is used to depict expression and degradation reactions. (PDF) [file pcbi.1007849.s001.pdf]

S1 Fig: Reaction network (full model)

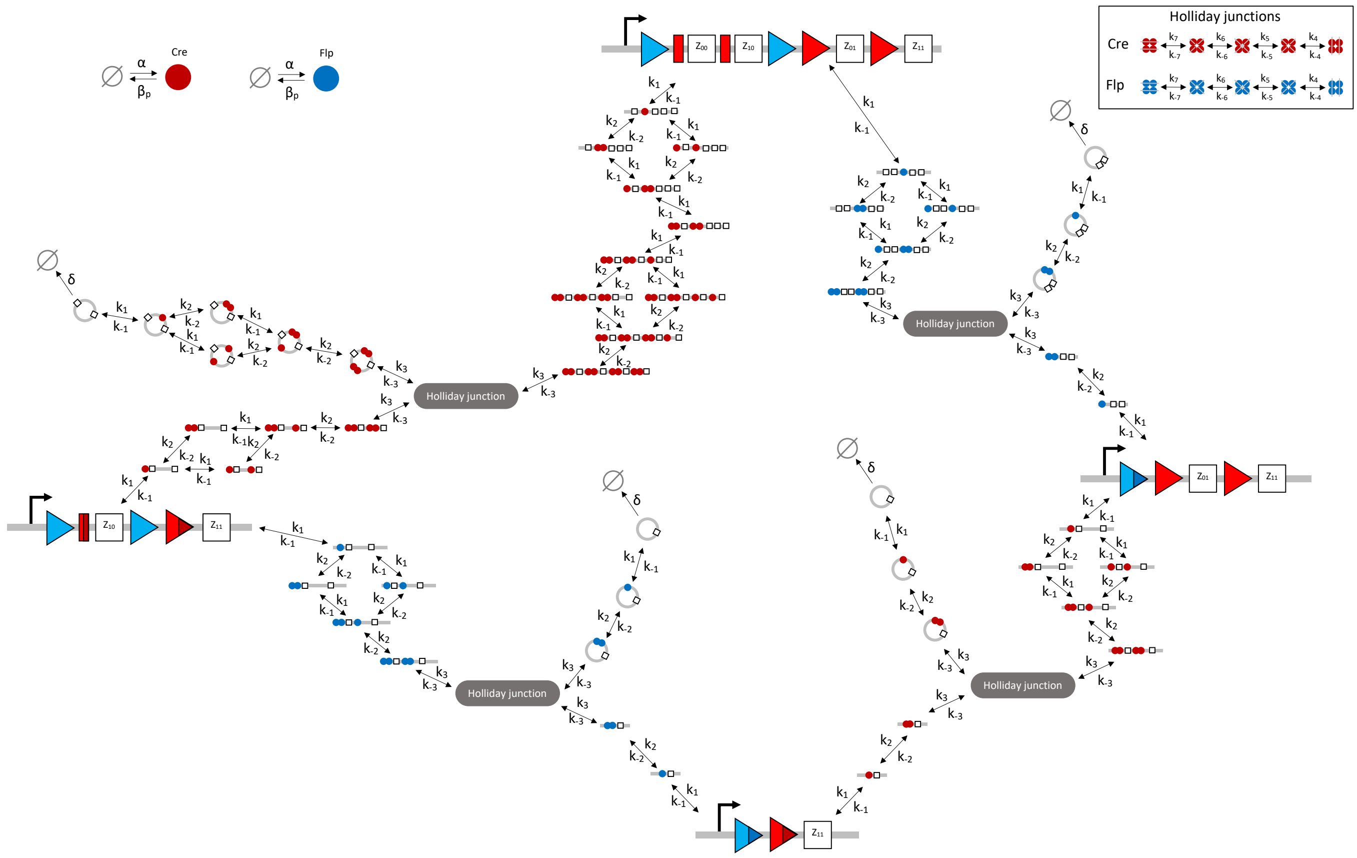

Figure 1: Schematic diagram of the tyrosine recombinase-mediated 2-input BLADE platform. Tyrosine recombinases Cre (red circles) and Flp (blue circles) are expressed constitutively at the same rate, α. LoxP sites are depicted as red triangles with different shades used to illustrate the result of recombination events. FRT sites are depicted as blue triangles with different shades used to illustrate the result of recombination events. White boxes depict each of the four BLADE addresses. Black arrows depict DNA:protein binding reactions comprising stable excision events. The rate of degradation of recombinase protein is denoted by β_p. The rate of a Cre/Flp monomer binding reversibly to free loxP/FRT sites is denoted by k_1, k_{-1}; due to the cooperativity of monomer binding, we denote the rate of a Cre/Flp monomer binding reversibly to an occupied loxP/FRT site by k_2, k_{-2}. The rate of Holliday junction formation is denoted by k_3, k_{-3}. Each of the five Holliday junction strand exchanges are denoted by k_4, k_{-4}, k_5, k_{-5}, k_6, k_{-6}, k_7, k_{-7}, respectively. The rate of dilution of excised DNA due to cell division is denoted by δ. The empty set symbol is used to depict expression and degradation reactions.
