# Supplementary material for: A mechanistic model of the BLADE platform predicts performance characteristics of 256 different synthetic DNA recombination circuits
Source: PLoS Comput Biol. 2020 Dec 18;16(12):e1007849. doi: 10.1371/journal.pcbi.1007849 (PMC7781486; doi:10.1371/journal.pcbi.1007849)
Supplement: S3 Fig — Histogram showing the distribution of Gillespie model simulations of the adapted metric scores for the 113 BLADE circuits tested experimentally in [8]. The mean and standard deviation are denoted by μ and σ respectively. The majority of circuits perform within 2° of optimal performance (0°). Gillespie simulations performed using the optimal parameter set identified through global optimisation. (PDF) [file pcbi.1007849.s003.pdf]

S3 Fig: Histogram

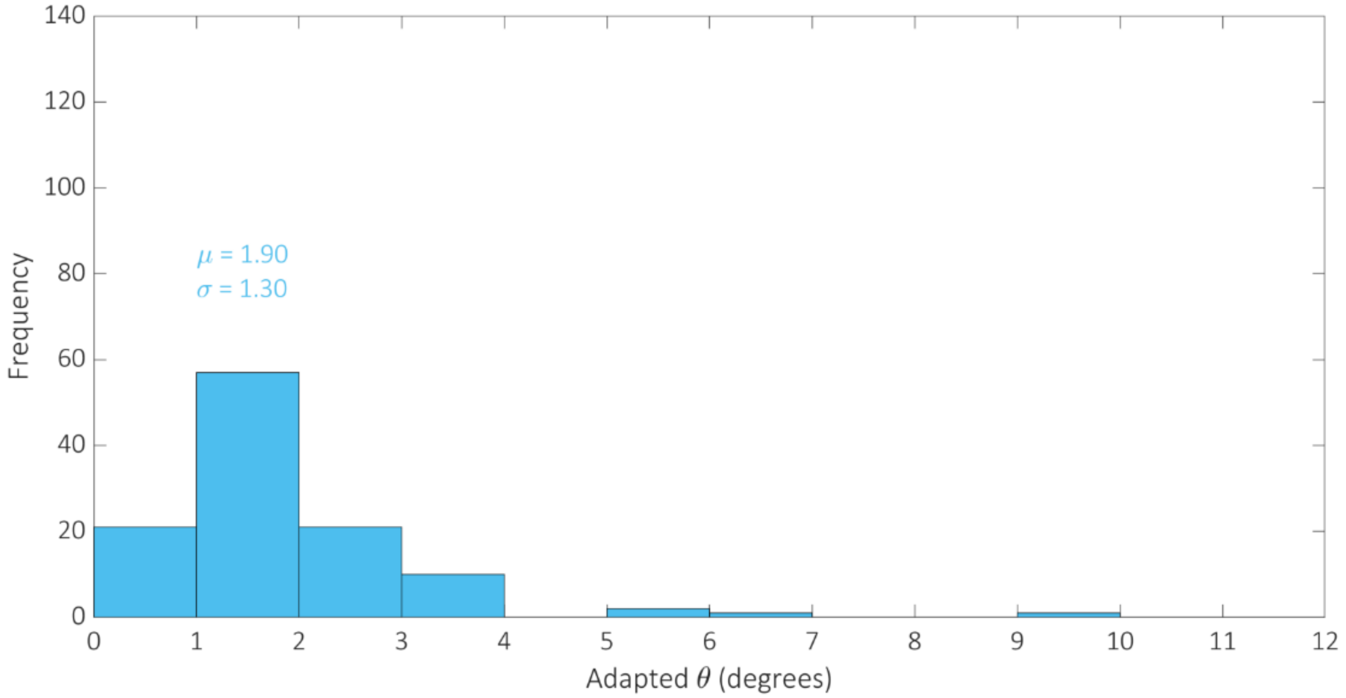

Figure 1: Histogram showing the distribution of Gillespie model simulations of the adapted metric scores for the 113 BLADE circuits tested experimentally in [8]. The mean and standard deviation are denoted by μ and σ respectively. The majority of circuits perform within 2° of optimal performance (0°). Gillespie simulations performed using the optimal parameter set identified through global optimisation.
