# Supplementary material for: A mechanistic model of the BLADE platform predicts performance characteristics of 256 different synthetic DNA recombination circuits
Source: PLoS Comput Biol. 2020 Dec 18;16(12):e1007849. doi: 10.1371/journal.pcbi.1007849 (PMC7781486; doi:10.1371/journal.pcbi.1007849)
Supplement: S4 Fig — Histograms showing the distributions of final population GA solutions for a separate run (blue). The orange bars are not distributions, but indicate the bin in which the corresponding optimal parameter value is located. (PDF) [file pcbi.1007849.s004.pdf]

S4 Fig: Histograms

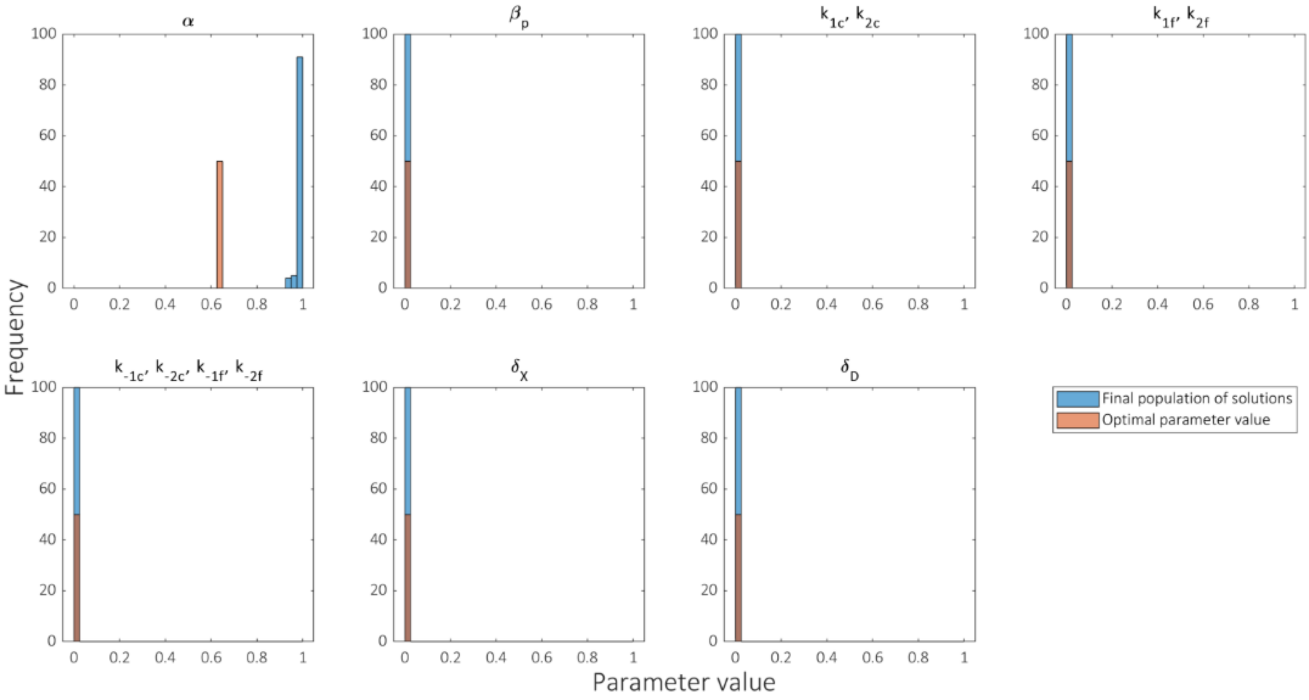

Figure 1: Histograms showing the distributions of final population GA solutions for a separate optimisation run (blue). The orange bars are not distributions, but indicate the bin in which the corresponding optimal parameter value is located; the bins match up for almost all parameters. Distinct optimisation runs provide similar results, and hence it is likely that the parameterisation we have identified is one of only a small number of similar parameterisations that provides the model outputs reported here.
