# Supplementary material for: A mechanistic model of the BLADE platform predicts performance characteristics of 256 different synthetic DNA recombination circuits
Source: PLoS Comput Biol. 2020 Dec 18;16(12):e1007849. doi: 10.1371/journal.pcbi.1007849 (PMC7781486; doi:10.1371/journal.pcbi.1007849)
Supplement: S1 Table — Biochemical equations describing the full reaction network of the 2-input BLADE platform (S1 Fig). Reactions are paired for the two separate Flp-mediated excision events for the sake of brevity i.e. one set of equations for both f1 and f2 (right column), since these two events consist of identical reactions. The left column lists the equivalent reactions that correspond to both Cre-mediated excision events, c1 and c2, however the middle column lists those reactions unique to c1, due to the fact that c1 consists of more reactions than c2 as well as f1 and f2. Cre and Flp that are expressed constitutively and mediate the four excision events are denoted by C and F, respectively. Cre and Flp monomers bind to DNA attachment sites sequentially until two monomers occupy each site; C1,1,F1,1 and C2,0,F2,0 denote one monomer bound to each site and two monomers bound to one site, respectively. DNA is denoted by D with a subscript corresponding to one of the four DNA states (D00, D10, D01, D11) or four excised DNA states (D00X, D10X, D01X, D11X), and with a superscript corresponding to one of the four recombination events (c1, c2, f1, f2). Each of the five strand exchanges that comprise a Holliday junction is denoted by the corresponding numbered H. (PDF) [file pcbi.1007849.s005.pdf]

S1 Table: Biochemical equations (full model)

$$\varnothing \underset{\beta_p}{\overset{\alpha}{\rightleftharpoons}} C$$

$$\varnothing \underset{\beta_p}{\overset{\alpha}{\rightleftharpoons}} F$$

$$D_{00,01} + C \underset{k_{-1}}{\overset{k_1}{\rightleftharpoons}} D^{c_1,c_2}_{00,01}C$$

$$D^{c_1,c_2}_{00,01}C + C \underset{k_{-2}}{\overset{k_2}{\rightleftharpoons}} D^{c_1,c_2}_{00,01}C_{2,0}$$

$$D^{c_1,c_2}_{00,01}C + C \underset{k_{-1}}{\overset{k_1}{\rightleftharpoons}} D^{c_1,c_2}_{00,01}C_{1,1}$$

$$D^{c_1,c_2}_{00,01}C_{2,0} + C \underset{k_{-1}}{\overset{k_1}{\rightleftharpoons}} D^{c_1,c_2}_{00,01}C_3$$

$$D^{c_1,c_2}_{00,01}C_{1,1} + C \underset{k_{-2}}{\overset{k_2}{\rightleftharpoons}} D^{c_1,c_2}_{00,01}C_3$$

$$D^{c_1,c_2}_{00,01}C_3 + C \underset{k_{-2}}{\overset{k_2}{\rightleftharpoons}} D^{c_1,c_2}_{00,01}C_4$$

$$D^{c_1}_{00}C_8, D^{c_2}_{01}C_4 \underset{k_{-3}}{\overset{k_3}{\rightleftharpoons}} H^{c_1,c_2}_1$$

$$H^{c_1,c_2}_1 \underset{k_{-4}}{\overset{k_4}{\rightleftharpoons}} H^{c_1,c_2}_2$$

$$H^{c_1,c_2}_2 \underset{k_{-5}}{\overset{k_5}{\rightleftharpoons}} H^{c_1,c_2}_3$$

$$H^{c_1,c_2}_3 \underset{k_{-6}}{\overset{k_6}{\rightleftharpoons}} H^{c_1,c_2}_4$$

$$H^{c_1,c_2}_4 \underset{k_{-7}}{\overset{k_7}{\rightleftharpoons}} H^{c_1,c_2}_5$$

$$D^{c_2}_{11}C_2 + D^{c_2}_{11_X}C_2 \underset{k_{-3}}{\overset{k_3}{\rightleftharpoons}} H^{c_2}_5$$

$$D^{c_2}_{11}C + C \underset{k_{-2}}{\overset{k_2}{\rightleftharpoons}} D^{c_2}_{11}C_2$$

$$D_{11} + C \underset{k_{-1}}{\overset{k_1}{\rightleftharpoons}} D^{c_2}_{11}C$$

$$D^{c_2}_{11_X}C + C \underset{k_{-2}}{\overset{k_2}{\rightleftharpoons}} D^{c_2}_{11_X}C_2$$

$$D^{c_2}_{11_X} + C \underset{k_{-1}}{\overset{k_1}{\rightleftharpoons}} D^{c_2}_{11_X}C$$

$$D^{c_2}_{11_X} \xrightarrow{\delta} \varnothing$$

$$D^{c_1}_{00}C_4 + C \underset{k_{-1}}{\overset{k_1}{\rightleftharpoons}} D^{c_1}_{00}C_5$$

$$D^{c_1}_{00}C_5 + C \underset{k_{-2}}{\overset{k_2}{\rightleftharpoons}} D^{c_1}_{00}C_{6,0}$$

$$D^{c_1}_{00}C_5 + C \underset{k_{-1}}{\overset{k_1}{\rightleftharpoons}} D^{c_1}_{00}C_{5,1}$$

$$D^{c_1}_{00}C_{6,0} + C \underset{k_{-1}}{\overset{k_1}{\rightleftharpoons}} D^{c_1}_{00}C_7$$

$$D^{c_1}_{00}C_{5,1} + C \underset{k_{-2}}{\overset{k_2}{\rightleftharpoons}} D^{c_1}_{00}C_7$$

$$D^{c_1}_{00}C_7 + C \underset{k_{-2}}{\overset{k_2}{\rightleftharpoons}} D^{c_1}_{00}C_8$$

$$D^{c_1}_{10}C_4 + D^{c_1}_{10_X}C_4 \underset{k_{-3}}{\overset{k_3}{\rightleftharpoons}} H^{c_1}_5$$

$$D^{c_1,c_1}_{10,10_X}C_3 + C \underset{k_{-2}}{\overset{k_2}{\rightleftharpoons}} D^{c_1,c_1}_{10,10_X}C_4$$

$$D^{c_1,c_1}_{10,10_X}C_{1,1} + C \underset{k_{-2}}{\overset{k_2}{\rightleftharpoons}} D^{c_1,c_1}_{10,10_X}C_3$$

$$D^{c_1,c_1}_{10,10_X}C_{2,0} + C \underset{k_{-1}}{\overset{k_1}{\rightleftharpoons}} D^{c_1,c_1}_{10,10_X}C_3$$

$$D^{c_1,c_1}_{10,10_X}C + C \underset{k_{-1}}{\overset{k_1}{\rightleftharpoons}} D^{c_1,c_1}_{10,10_X}C_{1,1}$$

$$D^{c_1,c_1}_{10,10_X}C + C \underset{k_{-2}}{\overset{k_2}{\rightleftharpoons}} D^{c_1,c_1}_{10,10_X}C_{2,0}$$

$$D_{10} + C \underset{k_{-1}}{\overset{k_1}{\rightleftharpoons}} D^{c_1}_{10}C$$

$$D^{c_1}_{10_X} + C \underset{k_{-1}}{\overset{k_1}{\rightleftharpoons}} D^{c_1}_{10_X}C$$

$$D^{c_1}_{10_X} \xrightarrow{\delta} \varnothing$$

$$D_{00,10} + F \underset{k_{-1}}{\overset{k_1}{\rightleftharpoons}} D^{f_1,f_2}_{00,10}F$$

$$D^{f_1,f_2}_{00,10}F + F \underset{k_{-2}}{\overset{k_2}{\rightleftharpoons}} D^{f_1,f_2}_{00,10}F_{2,0}$$

$$D^{f_1,f_2}_{00,10}F + F \underset{k_{-1}}{\overset{k_1}{\rightleftharpoons}} D^{f_1,f_2}_{00,10}F_{1,1}$$

$$D^{f_1,f_2}_{00,10}F_{2,0} + F \underset{k_{-1}}{\overset{k_1}{\rightleftharpoons}} D^{f_1,f_2}_{00,10}F_3$$

$$D^{f_1,f_2}_{00,10}F_{1,1} + F \underset{k_{-2}}{\overset{k_2}{\rightleftharpoons}} D^{f_1,f_2}_{00,10}F_3$$

$$D^{f_1,f_2}_{00,10}F_3 + F \underset{k_{-2}}{\overset{k_2}{\rightleftharpoons}} D^{f_1,f_2}_{00,10}F_4$$

$$D^{f_1,f_2}_{00,10}F_4 \underset{k_{-3}}{\overset{k_3}{\rightleftharpoons}} H^{f_1,f_2}_1$$

$$H^{f_1,f_2}_1 \underset{k_{-4}}{\overset{k_4}{\rightleftharpoons}} H^{f_1,f_2}_2$$

$$H^{f_1,f_2}_2 \underset{k_{-5}}{\overset{k_5}{\rightleftharpoons}} H^{f_1,f_2}_3$$

$$H^{f_1,f_2}_3 \underset{k_{-6}}{\overset{k_6}{\rightleftharpoons}} H^{f_1,f_2}_4$$

$$H^{f_1,f_2}_4 \underset{k_{-7}}{\overset{k_7}{\rightleftharpoons}} H^{f_1,f_2}_5$$

$$D^{f_1,f_2}_{10,11}F_2 + D^{f_1,f_2}_{01_X,11_X}F_2 \underset{k_{-3}}{\overset{k_3}{\rightleftharpoons}} H^{f_1,f_2}_5$$

$$D^{f_1,f_2}_{01,11}F + F \underset{k_{-2}}{\overset{k_2}{\rightleftharpoons}} D^{f_1,f_2}_{01,11}F_2$$

$$D_{01,11} + F \underset{k_{-1}}{\overset{k_1}{\rightleftharpoons}} D^{f_1,f_2}_{01,11}F$$

$$D^{f_1,f_2}_{01_X,11_X}F + F \underset{k_{-2}}{\overset{k_2}{\rightleftharpoons}} D^{f_1,f_2}_{01_X,11_X}F_2$$

$$D^{f_1,f_2}_{01_X,11_X} + F \underset{k_{-1}}{\overset{k_1}{\rightleftharpoons}} D^{f_1,f_2}_{01_X,11_X}F$$

$$D^{f_1}_{01_X} \xrightarrow{\delta} \varnothing$$

$$D^{f_2}_{11_X} \xrightarrow{\delta} \varnothing$$

Table 1: Biochemical equations describing the full reaction network of the 2-input BLADE platform (Fig. 2). Reactions are paired for the two separate Flp-mediated excision events for the sake of brevity i.e. one set of equations for both f_1 and f_2 (right column), since these two events consist of identical reactions. The left column lists the equivalent reactions that correspond to both Cre-mediated excision events, c_1 and c_2, however the middle column lists those reactions unique to c_1, due to the fact that c_1 consists of more reactions than c_2 as well as f_1 and f_2. Cre and Flp that are expressed constitutively and mediate the four excision events are denoted by C and F, respectively. Cre and Flp monomers bind to DNA attachment sites sequentially until two monomers occupy each site; $C_{1,1}$,$F_{1,1}$ and $C_{2,0}$,$F_{2,0}$ denote one monomer bound to each site and two monomers bound to one site, respectively. DNA is denoted by D with a subscript corresponding to one of the four DNA states (D_{00}, D_{10}, D_{01}, D_{11}) or four excised DNA states (D_{00_X}, D_{10_X}, D_{01_X}, D_{11_X}), and with a superscript corresponding to one of the four recombination events (c_1, c_2, f_1, f_2). Each of the five strand exchanges that comprise a Holliday junction is denoted by the corresponding numbered H.
