# Supplementary material for: A mechanistic model of the BLADE platform predicts performance characteristics of 256 different synthetic DNA recombination circuits
Source: PLoS Comput Biol. 2020 Dec 18;16(12):e1007849. doi: 10.1371/journal.pcbi.1007849 (PMC7781486; doi:10.1371/journal.pcbi.1007849)
Supplement: S2 Table — Mechanistic model ODEs derived from biochemical equations through the application of mass action kinetics. (PDF) [file pcbi.1007849.s006.pdf]

S2 Table: Model ODEs (full model)

$$
\begin{aligned}
\frac{d[C]}{dt} = {} & \alpha - \beta_p[C] + ... \\
& k_{-1}[D^{c1}_{00}C] - k_1[D_{00}][C] + k_{-2}[D^{c1}_{00}C_{2,0}] - k_2[D^{c1}_{00}C][C] + k_{-1}[D^{c1}_{00}C_{1,1}] - k_1[D^{c1}_{00}C][C] + k_{-1}[D^{c1}_{00}C_3] - k_1[D^{c1}_{00}C_{2,0}][C] + ... \\
& k_{-2}[D^{c1}_{00}C_3] - k_2[D^{c1}_{00}C_{1,1}][C] + k_{-2}[D^{c1}_{00}C_4] - k_2[D^{c1}_{00}C_3][C] + k_{-1}[D^{c1}_{00}C_5] - k_1[D^{c1}_{00}C_4][C] + k_{-2}[D^{c1}_{00}C_{6,0}] - k_2[D^{c1}_{00}C_5][C] + ... \\
& k_{-1}[D^{c1}_{00}C_{5,1}] - k_1[D^{c1}_{00}C_5][C] + k_{-1}[D^{c1}_{00}C_7] - k_1[D^{c1}_{00}C_{6,0}][C] + k_{-2}[D^{c1}_{00}C_7] - k_2[D^{c1}_{00}C_{5,1}][C] + k_{-2}[D^{c1}_{00}C_8] - k_2[D^{c1}_{00}C_7][C] + ... \\
& k_{-2}[D^{c1}_{10_X}C_4] - k_2[D^{c1}_{10_X}C_3][C] + k_{-2}[D^{c1}_{10_X}C_3] - k_2[D^{c1}_{10_X}C_{1,1}][C] + k_{-1}[D^{c1}_{10_X}C_3] - k_1[D^{c1}_{10_X}C_{2,0}][C] + ... \\
& k_{-2}[D^{c1}_{10_X}C_{2,0}] - k_2[D^{c1}_{10_X}C][C] + ... \\
& k_{-1}[D^{c1}_{10_X}C_{1,1}] - k_1[D^{c1}_{10_X}C][C] + k_{-1}[D^{c1}_{10_X}C] - k_1[D^{c1}_{10_X}][C] + k_{-2}[D^{c1}_{10}C_4] - k_2[D^{c1}_{10}C_3][C] + k_{-2}[D^{c1}_{10}C_3] - k_2[D^{c1}_{10}C_{1,1}][C] + ... \\
& k_{-1}[D^{c1}_{10}C_3] - k_1[D^{c1}_{10}C_{2,0}][C] + k_{-2}[D^{c1}_{10}C_{2,0}] - k_2[D^{c1}_{10}C][C] + k_{-1}[D^{c1}_{10}C_{1,1}] - k_1[D^{c1}_{10}C][C] + k_{-1}[D^{c1}_{10}C] - k_1[D_{10}][C] + ... \\
& k_{-1}[D^{c2}_{11}C] - k_1[D_{11}][C] + k_{-2}[D^{c2}_{01}C_{2,0}] - k_2[D^{c2}_{01}C][C] + k_{-1}[D^{c2}_{01}C_{1,1}] - k_1[D^{c2}_{01}C][C] + k_{-1}[D^{c2}_{01}C_3] - k_1[D^{c2}_{01}C_{2,0}][C] + ... \\
& k_{-2}[D^{c2}_{01}C_3] - k_2[D^{c2}_{01}C_{1,1}][C] + k_{-2}[D^{c2}_{01}C_4] - k_2[D^{c2}_{01}C_3][C] + k_{-2}[D^{c2}_{11_X}C_2] - k_2[D^{c2}_{11_X}C][C] + k_{-1}[D^{c2}_{11_X}C] - k_1[D^{c2}_{11_X}][C] + ... \\
& k_{-2}[D^{c2}_{11}C_2] - k_2[D^{c2}_{11}C][C] + k_{-1}[D^{c2}_{01}C] - k_1[D_{01}][C], \\
\frac{d[F]}{dt} = {} & \alpha - \beta_p[F] + ... \\
& k_{-1}[D^{f1}_{00}F] - k_1[D_{00}][F] + k_{-1}[D^{f2}_{10}F] - k_1[D_{10}][F] + k_{-2}[D^{f1}_{00}F_{2,0}] - k_2[D^{f1}_{00}F][F] + k_{-1}[D^{f1}_{00}F_{1,1}] - k_1[D^{f1}_{00}F][F] + ... \\
& k_{-1}[D^{f1}_{00}F_3] - k_1[D^{f1}_{00}F_{2,0}][F] + k_{-2}[D^{f1}_{00}F_3] - k_2[D^{f1}_{00}F_{1,1}][F] + k_{-2}[D^{f1}_{00}F_4] - k_2[D^{f1}_{00}F_3][F] + k_{-2}[D^{f1}_{01_X}F_2] - k_2[D^{f1}_{01_X}F][F] + ... \\
& k_{-1}[D^{f1}_{01_X}F] - k_1[D^{f1}_{01_X}][F] + k_{-2}[D^{f1}_{01}F_2] - k_2[D^{f1}_{01}F][F] + k_{-1}[D^{f1}_{01}F] - k_1[D_{01}][F] + k_{-2}[D^{f2}_{10}F_{2,0}] - k_2[D^{f2}_{10}F][F] + ... \\
& k_{-1}[D^{f2}_{10}F_{1,1}] - k_1[D^{f2}_{10}F][F] + k_{-1}[D^{f2}_{10}F_3] - k_1[D^{f2}_{10}F_{2,0}][F] + k_{-2}[D^{f2}_{10}F_3] - k_2[D^{f2}_{10}F_{1,1}][F] + k_{-2}[D^{f2}_{10}F_4] - k_2[D^{f2}_{10}F_3][F] + ... \\
& k_{-2}[D^{f2}_{11_X}F_2] - k_2[D^{f2}_{11_X}F][F] + k_{-1}[D^{f2}_{11_X}F] - k_1[D^{f2}_{11_X}][F] + k_{-2}[D^{f2}_{11}F_2] - k_2[D^{f2}_{11}F][F] + k_{-1}[D^{f2}_{11}F] - k_1[D_{11}][F], \\
\frac{d[D_{00}]}{dt} = {} & k_{-1}[D^{c1}_{00}C] - k_1[D_{00}][C] + k_{-1}[D^{f1}_{00}F] - k_1[D_{00}][F], \\
\frac{d[D^{c1}_{00}C]}{dt} = {} & k_1[D_{00}][C] - k_{-1}[D^{c1}_{00}C] + k_{-2}[D^{c1}_{00}C_{2,0}] - k_2[D^{c1}_{00}C][C] + k_{-1}[D^{c1}_{00}C_{1,1}] - k_1[D^{c1}_{00}C][C], \\
\frac{d[D^{c1}_{00}C_{2,0}]}{dt} = {} & k_2[D^{c1}_{00}C][C] - k_{-2}[D^{c1}_{00}C_{2,0}] + k_{-1}[D^{c1}_{00}C_3] - k_1[D^{c1}_{00}C_{2,0}][C], \\
\frac{d[D^{c1}_{00}C_{1,1}]}{dt} = {} & k_1[D^{c1}_{00}C][C] - k_{-1}[D^{c1}_{00}C_{1,1}] + k_{-2}[D^{c1}_{00}C_3] - k_2[D^{c1}_{00}C_{1,1}][C], \\
\frac{d[D^{c1}_{00}C_3]}{dt} = {} & k_1[D^{c1}_{00}C_{2,0}][C] - k_{-1}[D^{c1}_{00}C_3] + k_2[D^{c1}_{00}C_{1,1}][C] - k_{-2}[D^{c1}_{00}C_3] + k_{-2}[D^{c1}_{00}C_4] - k_2[D^{c1}_{00}C_3][C], \\
\frac{d[D^{c1}_{00}C_4]}{dt} = {} & k_2[D^{c1}_{00}C_3][C] - k_{-2}[D^{c1}_{00}C_4] + k_{-1}[D^{c1}_{00}C_5] - k_1[D^{c1}_{00}C_4][C], \\
\frac{d[D^{c1}_{00}C_5]}{dt} = {} & k_1[D^{c1}_{00}C_4][C] - k_{-1}[D^{c1}_{00}C_5] + k_{-2}[D^{c1}_{00}C_{6,0}] - k_2[D^{c1}_{00}C_5][C] + k_{-1}[D^{c1}_{00}C_{5,1}] - k_1[D^{c1}_{00}C_5][C], \\
\frac{d[D^{c1}_{00}C_{6,0}]}{dt} = {} & k_2[D^{c1}_{00}C_5][C] - k_{-2}[D^{c1}_{00}C_{6,0}] + k_{-1}[D^{c1}_{00}C_7] - k_1[D^{c1}_{00}C_{6,0}][C], \\
\frac{d[D^{c1}_{00}C_{5,1}]}{dt} = {} & k_1[D^{c1}_{00}C_5][C] - k_{-1}[D^{c1}_{00}C_{5,1}] + k_{-2}[D^{c1}_{00}C_7] - k_2[D^{c1}_{00}C_{5,1}][C], \\
\frac{d[D^{c1}_{00}C_7]}{dt} = {} & k_1[D^{c1}_{00}C_{6,0}][C] - k_{-1}[D^{c1}_{00}C_7] + k_2[D^{c1}_{00}C_{5,1}][C] - k_{-2}[D^{c1}_{00}C_7] + k_{-2}[D^{c1}_{00}C_8] - k_2[D^{c1}_{00}C_7][C], \\
\frac{d[D^{c1}_{00}C_8]}{dt} = {} & k_2[D^{c1}_{00}C_7][C] - k_{-2}[D^{c1}_{00}C_8] - k_3[D^{c1}_{00}C_8] + k_{-3}[H^{c1}_1], \\
\frac{d[H^{c1}_1]}{dt} = {} & k_3[D^{c1}_{00}C_8] - k_{-3}[H^{c1}_1] - k_4[H^{c1}_1] + k_{-4}[H^{c1}_2], \\
\frac{d[H^{c1}_2]}{dt} = {} & k_4[H^{c1}_1] - k_{-4}[H^{c1}_2] - k_5[H^{c1}_2] + k_{-5}[H^{c1}_3], \\
\frac{d[H^{c1}_3]}{dt} = {} & k_5[H^{c1}_2] - k_{-5}[H^{c1}_3] - k_6[H^{c1}_3] + k_{-6}[H^{c1}_4], \\
\frac{d[H^{c1}_4]}{dt} = {} & k_6[H^{c1}_3] - k_{-6}[H^{c1}_4] - k_7[H^{c1}_4] + k_{-7}[H^{c1}_5], \\
\frac{d[H^{c1}_5]}{dt} = {} & k_7[H^{c1}_4] - k_{-7}[H^{c1}_5] - k_{-3}[H^{c1}_5] + k_3[D^{c1}_{10_X}C_4][D^{c1}_{10}C_4], \\
\frac{d[D^{c1}_{10_X}C_4]}{dt} = {} & k_2[D^{c1}_{10_X}C_3][C] - k_{-2}[D^{c1}_{10_X}C_4] - k_3[D^{c1}_{10_X}C_4][D^{c1}_{10}C_4] + k_{-3}[H^{c1}_5], \\
\frac{d[D^{c1}_{10_X}C_3]}{dt} = {} & k_1[D^{c1}_{10_X}C_{2,0}][C] - k_{-1}[D^{c1}_{10_X}C_3] + k_2[D^{c1}_{10_X}C_{1,1}][C] - k_{-2}[D^{c1}_{10_X}C_3] + k_{-2}[D^{c1}_{10_X}C_4] - k_2[D^{c1}_{10_X}C_3][C], \\
\frac{d[D^{c1}_{10_X}C_{1,1}]}{dt} = {} & k_1[D^{c1}_{10_X}C][C] - k_{-1}[D^{c1}_{10_X}C_{1,1}] + k_{-2}[D^{c1}_{10_X}C_3] - k_2[D^{c1}_{10_X}C_{1,1}][C], \\
\frac{d[D^{c1}_{10_X}C_{2,0}]}{dt} = {} & k_2[D^{c1}_{10_X}C][C] - k_{-2}[D^{c1}_{10_X}C_{2,0}] + k_{-1}[D^{c1}_{10_X}C_3] - k_1[D^{c1}_{10_X}C_{2,0}][C], \\
\frac{d[D^{c1}_{10_X}C]}{dt} = {} & k_1[D^{c1}_{10_X}][C] - k_{-1}[D^{c1}_{10_X}C] + k_{-2}[D^{c1}_{10_X}C_{2,0}] - k_2[D^{c1}_{10_X}C][C] + k_{-1}[D^{c1}_{10_X}C_{1,1}] - k_1[D^{c1}_{10_X}C][C], \\
\frac{d[D^{c1}_{10_X}]}{dt} = {} & k_{-1}[D^{c1}_{10_X}C] - k_1[D^{c1}_{10_X}][C] - \delta[D^{c1}_{10_X}], \\
\frac{d[D^{c1}_{10}C_4]}{dt} = {} & k_2[D^{c1}_{10}C_3][C] - k_{-2}[D^{c1}_{10}C_4] - k_3[D^{c1}_{10_X}C_4][D^{c1}_{10}C_4] + k_{-3}[H^{c1}_5], \\
\frac{d[D^{c1}_{10}C_3]}{dt} = {} & k_1[D^{c1}_{10}C_{2,0}][C] - k_{-1}[D^{c1}_{10}C_3] + k_2[D^{c1}_{10}C_{1,1}][C] - k_{-2}[D^{c1}_{10}C_3] + k_{-2}[D^{c1}_{10}C_4] - k_2[D^{c1}_{10}C_3][C], \\
\frac{d[D^{c1}_{10}C_{1,1}]}{dt} = {} & k_1[D^{c1}_{10}C][C] - k_{-1}[D^{c1}_{10}C_{1,1}] + k_{-2}[D^{c1}_{10}C_3] - k_2[D^{c1}_{10}C_{1,1}][C], \\
\frac{d[D^{c1}_{10}C_{2,0}]}{dt} = {} & k_2[D^{c1}_{10}C][C] - k_{-2}[D^{c1}_{10}C_{2,0}] + k_{-1}[D^{c1}_{10}C_3] - k_1[D^{c1}_{10}C_{2,0}][C], \\
\frac{d[D^{c1}_{10}C]}{dt} = {} & k_1[D_{10}][C] - k_{-1}[D^{c1}_{10}C] + k_{-2}[D^{c1}_{10}C_{2,0}] - k_2[D^{c1}_{10}C][C] + k_{-1}[D^{c1}_{10}C_{1,1}] - k_1[D^{c1}_{10}C][C], \\
\frac{d[D_{10}]}{dt} = {} & k_{-1}[D^{c1}_{10}C] - k_1[D_{10}][C] + k_{-1}[D^{f2}_{10}F] - k_1[D_{10}][F], \\
\frac{d[D^{f1}_{00}F]}{dt} = {} & k_1[D_{00}][F] - k_{-1}[D^{f1}_{00}F] + k_{-2}[D^{f1}_{00}F_{2,0}] - k_2[D^{f1}_{00}F][F] + k_{-1}[D^{f1}_{00}F_{1,1}] - k_1[D^{f1}_{00}F][F], \\
\frac{d[D^{f1}_{00}F_{2,0}]}{dt} = {} & k_2[D^{f1}_{00}F][F] - k_{-2}[D^{f1}_{00}F_{2,0}] + k_{-1}[D^{f1}_{00}F_3] - k_1[D^{f1}_{00}F_{2,0}][F], \\
\frac{d[D^{f1}_{00}F_{1,1}]}{dt} = {} & k_1[D^{f1}_{00}F][F] - k_{-1}[D^{f1}_{00}F_{1,1}] + k_{-2}[D^{f1}_{00}F_3] - k_2[D^{f1}_{00}F_{1,1}][F], \\
\frac{d[D^{f1}_{00}F_3]}{dt} = {} & k_1[D^{f1}_{00}F_{2,0}][F] - k_{-1}[D^{f1}_{00}F_3] + k_2[D^{f1}_{00}F_{1,1}][F] - k_{-2}[D^{f1}_{00}F_3] + k_{-2}[D^{f1}_{00}F_4] - k_2[D^{f1}_{00}F_3][F], \\
\frac{d[D^{f1}_{00}F_4]}{dt} = {} & k_2[D^{f1}_{00}F_3][F] - k_{-2}[D^{f1}_{00}F_4] - k_3[D^{f1}_{00}F_4] + k_{-3}[H^{f1}_1], \\
\frac{d[H^{f1}_1]}{dt} = {} & k_3[D^{f1}_{00}F_4] - k_{-3}[H^{f1}_1] - k_4[H^{f1}_1] + k_{-4}[H^{f1}_2], \\
\frac{d[H^{f1}_2]}{dt} = {} & k_4[H^{f1}_1] - k_{-4}[H^{f1}_2] - k_5[H^{f1}_2] + k_{-5}[H^{f1}_3], \\
\frac{d[H^{f1}_3]}{dt} = {} & k_5[H^{f1}_2] - k_{-5}[H^{f1}_3] - k_6[H^{f1}_3] + k_{-6}[H^{f1}_4], \\
\frac{d[H^{f1}_4]}{dt} = {} & k_6[H^{f1}_3] - k_{-6}[H^{f1}_4] - k_7[H^{f1}_4] + k_{-7}[H^{f1}_5], \\
\frac{d[H^{f1}_5]}{dt} = {} & k_7[H^{f1}_4] - k_{-7}[H^{f1}_5] - k_{-3}[H^{f1}_5] + k_3[D^{f1}_{01_X}F_2][D^{f1}_{01}F_2],
\end{aligned}
$$

$$
\begin{aligned}
\frac{d[D^{f_1}_{01_X}F_2]}{dt} &= k_{-3}[H^{f_1}_5] - k_3[D^{f_1}_{01_X}F_2][D^{f_1}_{01}F_2] - k_{-2}[D^{f_1}_{01_X}F_2] + k_2[D^{f_1}_{01_X}F][F],\\
\frac{d[D^{f_1}_{01_X}F]}{dt} &= k_1[D^{f_1}_{01_X}][F] - k_{-1}[D^{f_1}_{01_X}F] + k_{-2}[D^{f_1}_{01_X}F_2] - k_2[D^{f_1}_{01_X}F][F],\\
\frac{d[D^{f_1}_{01_X}]}{dt} &= k_{-1}[D^{f_1}_{01_X}F] - k_1[D^{f_1}_{01_X}][F] - \delta[D^{f_1}_{01_X}],\\
\frac{d[D^{f_1}_{01}F_2]}{dt} &= k_{-3}[H^{f_1}_5] - k_3[D^{f_1}_{01_X}F_2][D^{f_1}_{01}F_2] - k_{-2}[D^{f_1}_{01}F_2] + k_2[D^{f_1}_{01}F][F],\\
\frac{d[D^{f_1}_{01}F]}{dt} &= k_1[D_{01}][F] - k_{-1}[D^{f_1}_{01}F] + k_{-2}[D^{f_1}_{01}F_2] - k_2[D^{f_1}_{01}F][F],\\
\frac{d[D_{01}]}{dt} &= k_{-1}[D^{f_1}_{01}F] - k_1[D_{01}][F] + k_{-1}[D^{c_2}_{01}C] - k_1[D_{01}][C],\\
\frac{d[D^{f_2}_{10}F]}{dt} &= k_1[D_{10}][F] - k_{-1}[D^{f_2}_{10}F] + k_{-2}[D^{f_2}_{10}F_{2,0}] - k_2[D^{f_2}_{10}F][F] + k_{-1}[D^{f_2}_{10}F_{1,1}] - k_1[D^{f_2}_{10}F][F],\\
\frac{d[D^{f_2}_{10}F_{2,0}]}{dt} &= k_2[D^{f_2}_{10}F][F] - k_{-2}[D^{f_2}_{10}F_{2,0}] + k_{-1}[D^{f_2}_{10}F_3] - k_1[D^{f_2}_{10}F_{2,0}][F],\\
\frac{d[D^{f_2}_{10}F_{1,1}]}{dt} &= k_1[D^{f_2}_{10}F][F] - k_{-1}[D^{f_2}_{10}F_{1,1}] + k_{-2}[D^{f_2}_{10}F_3] - k_2[D^{f_2}_{10}F_{1,1}][F],\\
\frac{d[D^{f_2}_{10}F_3]}{dt} &= k_1[D^{f_2}_{10}F_{2,0}][F] - k_{-1}[D^{f_2}_{10}F_3] + k_2[D^{f_2}_{10}F_{1,1}][F] - k_{-2}[D^{f_2}_{10}F_3] + k_{-2}[D^{f_2}_{10}F_4] - k_2[D^{f_2}_{10}F_3][F],\\
\frac{d[D^{f_2}_{10}F_4]}{dt} &= k_2[D^{f_2}_{10}F_3][F] - k_{-2}[D^{f_2}_{10}F_4] - k_3[D^{f_2}_{10}F_4] + k_{-3}[H^{f_2}_1],\\
\frac{d[H^{f_2}_1]}{dt} &= k_3[D^{f_2}_{10}F_4] - k_{-3}[H^{f_2}_1] - k_4[H^{f_2}_1] + k_{-4}[H^{f_2}_2],\\
\frac{d[H^{f_2}_2]}{dt} &= k_4[H^{f_2}_1] - k_{-4}[H^{f_2}_2] - k_5[H^{f_2}_2] + k_{-5}[H^{f_2}_3],\\
\frac{d[H^{f_2}_3]}{dt} &= k_5[H^{f_2}_2] - k_{-5}[H^{f_2}_3] - k_6[H^{f_2}_3] + k_{-6}[H^{f_2}_4],\\
\frac{d[H^{f_2}_4]}{dt} &= k_6[H^{f_2}_3] - k_{-6}[H^{f_2}_4] - k_7[H^{f_2}_4] + k_{-7}[H^{f_2}_5],\\
\frac{d[H^{f_2}_5]}{dt} &= k_7[H^{f_2}_4] - k_{-7}[H^{f_2}_5] - k_{-3}[H^{f_2}_5] + k_3[D^{f_2}_{11_X}F_2][D^{f_2}_{11}F_2],\\
\frac{d[D^{f_2}_{11_X}F_2]}{dt} &= k_{-3}[H^{f_2}_5] - k_3[D^{f_2}_{11_X}F_2][D^{f_2}_{11}F_2] - k_{-2}[D^{f_2}_{11_X}F_2] + k_2[D^{f_2}_{11_X}F][F],\\
\frac{d[D^{f_2}_{11_X}F]}{dt} &= k_1[D^{f_2}_{11_X}][F] - k_{-1}[D^{f_2}_{11_X}F] + k_{-2}[D^{f_2}_{11_X}F_2] - k_2[D^{f_2}_{11_X}F][F],\\
\frac{d[D^{f_2}_{11_X}]}{dt} &= k_{-1}[D^{f_2}_{11_X}F] - k_1[D^{f_2}_{11_X}][F] - \delta[D^{f_2}_{11_X}],\\
\frac{d[D^{f_2}_{11}F_2]}{dt} &= k_{-3}[H^{f_2}_5] - k_3[D^{f_2}_{11_X}F_2][D^{f_2}_{11}F_2] - k_{-2}[D^{f_2}_{11}F_2] + k_2[D^{f_2}_{11}F][F],\\
\frac{d[D^{f_2}_{11}F]}{dt} &= k_1[D_{11}][F] - k_{-1}[D^{f_2}_{11}F] + k_{-2}[D^{f_2}_{11}F_2] - k_2[D^{f_2}_{11}F][F],\\
\frac{d[D_{11}]}{dt} &= k_{-1}[D^{f_2}_{11}F] - k_1[D_{11}][F] + k_{-1}[D^{c_2}_{11}C] - k_1[D_{11}][C],\\
\frac{d[D^{c_2}_{01}C]}{dt} &= k_1[D_{01}][C] - k_{-1}[D^{c_2}_{01}C] + k_{-2}[D^{c_2}_{01}C_{2,0}] - k_2[D^{c_2}_{01}C][C] + k_{-1}[D^{c_2}_{01}C_{1,1}] - k_1[D^{c_2}_{01}C][C],\\
\frac{d[D^{c_2}_{01}C_{2,0}]}{dt} &= k_2[D^{c_2}_{01}C][C] - k_{-2}[D^{c_2}_{01}C_{2,0}] + k_{-1}[D^{c_2}_{01}C_3] - k_1[D^{c_2}_{01}C_{2,0}][C],\\
\frac{d[D^{c_2}_{01}C_{1,1}]}{dt} &= k_1[D^{c_2}_{01}C][C] - k_{-1}[D^{c_2}_{01}C_{1,1}] + k_{-2}[D^{c_2}_{01}C_3] - k_2[D^{c_2}_{01}C_{1,1}][C],\\
\frac{d[D^{c_2}_{01}C_3]}{dt} &= k_1[D^{c_2}_{01}C_{2,0}][C] - k_{-1}[D^{c_2}_{01}C_3] + k_2[D^{c_2}_{01}C_{1,1}][C] - k_{-2}[D^{c_2}_{01}C_3] + k_{-2}[D^{c_2}_{01}C_4] - k_2[D^{c_2}_{01}C_3][C],\\
\frac{d[D^{c_2}_{01}C_4]}{dt} &= k_2[D^{c_2}_{01}C_3][C] - k_{-2}[D^{c_2}_{01}C_4] - k_3[D^{c_2}_{01}C_4] + k_{-3}[H^{c_2}_1],\\
\frac{d[H^{c_2}_1]}{dt} &= k_3[D^{c_2}_{01}C_4] - k_{-3}[H^{c_2}_1] - k_4[H^{c_2}_1] + k_{-4}[H^{c_2}_2],\\
\frac{d[H^{c_2}_2]}{dt} &= k_4[H^{c_2}_1] - k_{-4}[H^{c_2}_2] - k_5[H^{c_2}_2] + k_{-5}[H^{c_2}_3],\\
\frac{d[H^{c_2}_3]}{dt} &= k_5[H^{c_2}_2] - k_{-5}[H^{c_2}_3] - k_6[H^{c_2}_3] + k_{-6}[H^{c_2}_4],\\
\frac{d[H^{c_2}_4]}{dt} &= k_6[H^{c_2}_3] - k_{-6}[H^{c_2}_4] - k_7[H^{c_2}_4] + k_{-7}[H^{c_2}_5],\\
\frac{d[H^{c_2}_5]}{dt} &= k_7[H^{c_2}_4] - k_{-7}[H^{c_2}_5] - k_{-3}[H^{c_2}_5] + k_3[D^{c_2}_{11_X}C_2][D^{c_2}_{11}C_2],\\
\frac{d[D^{c_2}_{11_X}C_2]}{dt} &= k_{-3}[H^{c_2}_5] - k_3[D^{c_2}_{11_X}C_2][D^{c_2}_{11}C_2] - k_{-2}[D^{c_2}_{11_X}C_2] + k_2[D^{c_2}_{11_X}C][C],\\
\frac{d[D^{c_2}_{11_X}C]}{dt} &= k_1[D^{c_2}_{11_X}][C] - k_{-1}[D^{c_2}_{11_X}C] + k_{-2}[D^{c_2}_{11_X}C_2] - k_2[D^{c_2}_{11_X}C][C],\\
\frac{d[D^{c_2}_{11_X}]}{dt} &= k_{-1}[D^{c_2}_{11_X}C] - k_1[D^{c_2}_{11_X}][C] - \delta[D^{c_2}_{11_X}],\\
\frac{d[D^{c_2}_{11}C_2]}{dt} &= k_{-3}[H^{c_2}_5] - k_3[D^{c_2}_{11_X}C_2][D^{c_2}_{11}C_2] - k_{-2}[D^{c_2}_{11}C_2] + k_2[D^{c_2}_{11}C][C],\\
\frac{d[D^{c_2}_{11}C]}{dt} &= k_1[D_{11}][C] - k_{-1}[D^{c_2}_{11}C] + k_{-2}[D^{c_2}_{11}C_2] - k_2[D^{c_2}_{11}C][C].
\end{aligned}
$$

Table 1: Mechanistic model ODEs derived from biochemical equations through the application of mass action kinetics.
