# Supplementary material for: A mechanistic model of the BLADE platform predicts performance characteristics of 256 different synthetic DNA recombination circuits
Source: PLoS Comput Biol. 2020 Dec 18;16(12):e1007849. doi: 10.1371/journal.pcbi.1007849 (PMC7781486; doi:10.1371/journal.pcbi.1007849)
Supplement: S1 Text — (PDF) [file pcbi.1007849.s007.pdf]

S1 Text: Model simulations (full model)

In order to simulate the time course concentration dynamics of each address, we determine the total concentration of the system across the molecular complexes associated with each of these states. This involves summing the model ODEs that correspond to associated molecular entities, and hence we derive the ODEs governing the rate of change in concentration of each BLADE address:

$$\begin{aligned}
\frac{d[Z_{00}]}{dt} &= k_{-3}([H_1^{c_1}] + [H_1^{f_1}]) - k_3([D_{00}^{c_1}C_8] + [D_{00}^{f_1}F_4]),\\
\frac{d[Z_{10}]}{dt} &= k_{-3}([H_5^{c_1}] + [H_1^{f_2}]) - k_3([D_{10_X}^{c_1}C_4][D_{10}^{c_1}C_4] + [D_{10}^{f_2}F_4]),\\
\frac{d[Z_{01}]}{dt} &= k_{-3}([H_5^{f_1}] + [H_1^{c_2}]) - k_3([D_{01_X}^{f_1}F_2][D_{01}^{f_1}F_2] + [D_{01}^{c_2}C_4]),\\
\frac{d[Z_{11}]}{dt} &= k_{-3}([H_5^{f_2}] + [H_5^{c_2}]) - k_3([D_{11_X}^{f_2}F_2][D_{11}^{f_2}F_2] + [D_{11_X}^{c_2}C_2][D_{11}^{c_2}C_2]),
\end{aligned}$$

where D, H, C and F denote DNA, Holliday junction, Cre and Flp respectively. The parameters k_3 and k_{-3} are the forward and backward reactions rate constants corresponding to the formation of the first or fifth Holliday junction complex. Numerical subscripts denote the number of monomers, the address or specific Holliday junction complex (out of five). The numerical solutions to these four equations provide the outputs for the mechanistic model simulations of the BLADE platform.
