# Supplementary material for: A mechanistic model of the BLADE platform predicts performance characteristics of 256 different synthetic DNA recombination circuits
Source: PLoS Comput Biol. 2020 Dec 18;16(12):e1007849. doi: 10.1371/journal.pcbi.1007849 (PMC7781486; doi:10.1371/journal.pcbi.1007849)
Supplement: Attachment — Submitted filename: Reviewer_responses.docx [file pcbi.1007849.s008.docx]

Comments by reviewers are in **bold face**, our response is in *italics*.

**Reviewers responses to questions**

**Comments to the Authors:**

**Reviewer #1:**

**1. Applications**

**To be relevant to a broad audience, including the readers of PLOS CompBio, it would be helpful to motivate the need for the BLADE platform with two or three compelling examples of how it could or would be used.**

*We thank the reviewer for this helpful suggestion and have added the following text to the Introduction (page 2):*

*“The BLADE platform offers a robust and convenient framework for producing multi-input-multi-output circuits. These circuits can be used for tissue engineering applications, whereby different genes can be specifically expressed in different tissues. Similarly, BLADE is particularly useful in advanced animal model development where, again, one can express different genes in different tissues. For instance, it is possible to express two different inducible kill switches in order to kill off different cell types at different times to analyse their impact on development. Additionally, recombinase-based circuits can be used in immune cells for cancer diagnostics. When combined with tumour-specific sensors such as synNotch receptors, these circuits can provide combinatorial detection of antigens on cancer cells for highly specific tumour detection based on multiple tumour markers."*

**2. Complex vs. simplified models**

**It seems the simplified model (Fig 6) was the basis for stochastic simulations and evaluation of the model-vs-experiments. The full complex model (Fig 2) is useful to show for completeness, but please consider moving it to Supplementary information. As a general reader, I wasted a lot of time trying to decipher its meaning before discovering Fig 6, which is more transparent.**

*The figure of the reaction network associated with the full model (Fig. 2) has been moved to the Supplementary Information (Figure S1) as requested.*

**3. Practical issues**

**In the context of applications, how important is it that the cells be readily transfectable? Are the anticipated applications for cell lines rather than primary cells? How sensitive is the output to the 48h post-transfection period and the selection of the top 0.1 percent of transfection positive cells for quantitative analysis? I realise these are not central points to the paper. However, if your wish to engage the broad PLOS CompBio audience in potential real-world applications, I would recommend such issues at least be discussed.**

*In an earlier paper [8], we have shown that BLADE circuits can be stably integrated into the genome in human T cells. However, transient transfection is the most reasonable way to evaluate >100 synthetic circuits in human cells. This point has been clarified in the revised manuscript (Materials and Methods).*

**p.10 Equations 1-4 need to be numbered.**

*These equations have now been moved to the Supplementary Material (Section S1).*

**Reviewer #2**

**The paper presents results from a model of the Boolean logic and arithmetic through DNA excision (BLADE) genetic design platform. BLADE is a genetic design technique for mammalian cells that uses Cre and Flp-mediated DNA incision to turn on gates by inverting DNA to activate a gene or removing a terminator to activate a gene. Using this technique, one can construct combinational genetic logic gates. One important point that was not emphasized in the paper is that these are not exactly combinational logic gates in that they have state. Once the gate has changed state, they are typically not able to change state again with a change inputs as required to be a true combinational logic gate. The authors develop both an ODE and stochastic model and compare their results with experimental results for 111 circuits. They also make predictions about performance for an additional 143 circuits that have yet to be experimentally verified.**

*We agree with the reviewer and have added the following comment to the Introduction (page 2), to clarify the exact nature of the logic gates constructed via the BLADE platform:*

*“These circuits are Boolean in the sense that they provide outputs in response to TRUE/FALSE input conditions, however, unlike conventional logic gate circuits, once a state has been induced it is fixed and cannot be altered via subsequent input alterations.”*

**While the paper is generally well written and the model appears to be useful, a number of important details are omitted making it difficult to fully judge the model and reproduce any of the results. While a detailed ODE model is presented in the supplemental, the parameters are not provided. Parameters are only provided for a simplified model that I could not find in the paper or the supplemental. There is a simplified ODE model in the paper, but it refers to a k3 and k-3 parameters that I could not find. The stochastic model is even less detailed. There is a biochemical reaction model in the supplemental, but it does not appear that that is what they simulated using the Gillespie algorithm. The authors also do not mention what software they used for their analysis. All of these problems could be addressed by simply providing the model in electronic form (preferably in SBML), as well as instructions for running the model in the software they used. If they wrote their own software, this should also be released using github or similar. Until this information is released it is impossible to make a complete assessment of their results.**

*We agree with the reviewer that greater clarity is required:*

**While a detailed ODE model is presented in the supplemental, the parameters are not provided.**

*The parameters for the detailed ODE model are not provided because this model was not fit to the data and hence there are no “optimal” parameter values relating to the model. We have added the following comment to page 9 to clarify this point:*

*“Note that henceforth all results are generated using the simplified model (Figure 6) and the Gillespie algorithm simulations it is subjected to.”*

**Parameters are only provided for a simplified model that I could not find in the paper or the supplemental.**

*The ODEs describing the simplified model have been added to the main text and the associated optimal parameter values inferred through global optimisation have also been added to the main text in Table 3.*

**There is a simplified ODE model in the paper, but it refers to a k3 and k-3 parameters that I could not find.**

*We apologise for the confusion. This is not a simplified model, these ODEs describe the output of the BLADE platform relating to each of the four addresses accessible through the four Boolean inputs. The ODEs are derived from the detailed model ODEs (not the simplified model), through summation of the ODEs associated with molecular entities belonging to the same BLADE state. The parameters k_3_ and k_-3_ can be found in the detailed reaction network diagram that has now been moved to the Supplementary Information (Figure S1). To avoid confusion we have moved these ODE’s to the SI, where they now appear alongside the description of the detailed model, and focussed the exposition in the main paper on the simplified model.*

**The stochastic model is even less detailed. There is a biochemical reaction model in the supplemental, but it does not appear that that is what they simulated using the Gillespie algorithm.**

*The simplified model which was simulated using the Gillespie algorithm can be found in Fig. 6 and the ODEs have now been added to main paper. The following comment has been added to the Fig. 6 caption on page 25 to clarify this point:*

*“The simplified network consists of a reduced number of state variables and parameters in order to minimise the computational workload of running Gillespie algorithm simulations”*

**The authors also do not mention what software they used for their analysis. All of these problems could be addressed by simply providing the model in electronic form (preferably in SBML), as well as instructions for running the model in the software they used.**

*The relevant code can be found on GitHub (https://github.com/jackbowyer/BLADE_model). The following comments have been added to page 16 to clarify this point:*

*“We employed MATLAB R2018a to run Gillespie algorithm simulations, and have uploaded this MATLAB code along with an SBML version of our simplified model to GitHub.”*

*“The MATLAB code used to generate our results can be found on GitHub.”*

**Reviewer #3:**

**Bower et al. developed mechanistic model based on biochemical mass action kinetics to describe Flp- and Cre- induced DNA recombination in 256 circuits constructed out of a 2-input and 2-output BLADE platform. The authors use published and new data to estimate parameters in their mechanistic model and test model predictions. The mechanistic model is able to describe fluorescence data obtained from several circuits well and the trained models were used to generate predictions in other circuits; some of which were investigated in published experiments. The main conclusion from the study is that the circuit performance decreases with increasing number of STOP sequences and low number of recombinase molecules. I think the authors address an important problem of developing mechanistic computational models to describe Boolean synthetic biology circuits. They carry out a detailed investigation of their models to arrive at somewhat straightforward conclusions. In addition, there are few major shortcomings in the present work: 1) It is not clear how well the model parameters can be estimated. 2) The models and the estimated parameters were not used to glean any mechanisms that underlie DNA recombination. In addition, the text should be revised at several places to better describe their system, approach, and results. I provide further details regarding these issues below.

Major comments

1. The kinetic rates used in the model are estimated by minimizing a cost function using genetic algorithm. It is not clear how well the parameters described the data, for example, whether there are parameters with large confidence intervals or there are non-identifiable parameters. In the absence of that it makes it difficult to assess if the models are well parameterized. It also makes it difficult to investigate potential sources for the large differences between the model prediction and the data (e.g., Fig. 9 and Fig. 12). Though the qualitative trends somewhat agree in Fig. 12, for a model that is fitted well with the training data, it is unclear why the gap between the model prediction and the data will be this large. Perhaps, this disagreement points to identifiability issues in the current parameterization and/or the existence of alternate models?**

*The task of optimising the model using the genetic algorithm is complex in our case since we look to optimise an ensemble of Gillespie model simulations. Typically, there is a one-to-one correspondence between candidate parameterisations examined by the GA and their cost function output, however, this is not the case here as we attempt to identify an optimal parameterisation that provides consistent model output despite Gillespie simulations being inherently stochastic. In order to illustrate the confidence intervals of our optimal parameterisation, we have added a figure to the Supplementary Material. The figure consists of histograms of the distributions of final population GA solutions for a separate run to the run that identified our optimal parameterisation. The histogram bin containing the optimal parameter value is also added, and it can be seen that the bins match up for almost all parameters. This shows that distinct optimisation runs provide similar results, and hence it is likely that the parameterisation we have identified is one of only a small number of similar parameterisations that provides the model outputs reported here. We have added reference to this new figure in the main text. While the reviewer is correct that the model does not replicate the data exactly, this is not surprising given that the model is a significantly simplified version of that which includes all mechanistic features of the system (see the Supplementary Material). This simplification is, however, essential, in order to allow stochastic simulations. Finally, we would add that the purpose of our modelling here is not to identify “actual” values of kinetic rates, per se, but to produce a predictive simulation tool that can be useful in informing experimental work – we believe that the results presented in the paper demonstrate that this has been achieved.*

**2. The authors make no attempt to extract any mechanism from the estimated parameters or by generating results under a condition that can be informative about the underlying kinetics. The two main conclusions appear to be somewhat straightforward. For example, the conclusion that increasing number of STOP sequences lead to circuit lower performance probably can be inferred by systematic analysis of the published fluorescent data. As for the other conclusion, the lower performance of at the low copy numbers of recombinases could possibly arise due to the increase in the intrinsic noise fluctuations in the chemical kinetics which is kind of expected. Thus, it might be worthwhile to further analyze the models for obtaining non-intuitive/new mechanisms.**

*We appreciate the reviewer’s observations but would make a number of points in response. Firstly, the complexity of the system under investigation here, and the fact that it requires stochastic simulations, makes the extraction of insights into fundamental mechanisms inherently difficult. Secondly, this was not the primary objective of our modelling investigation – rather our aim was to develop a simulation tool that could predict the performance of different BLADE circuits in order to reduce the requirement for exhaustively constructing each one experimentally, and to generate insights into how to optimise their performance. We believe that it is noteworthy in and of itself that a single computational model can be constructed that can predict the performance of 256 different synthetic circuits. Finally, the insight that increasing number of STOP sequences lead to circuit lower performance was not obvious to our experimental collaborators who developed the BLADE system, and represents valuable new knowledge generated by our model.*

**3. I was hoping to find some mechanistic reasons behind the low circuit performance in experiments. Does intrinsic noise fluctuation have a role to play here or there are biological variables, not accounted for in the experiments (and in the model), result in the difference between the truth table and a circuit output? The authors also mention co-operativity in recombinase binding, could this co-operativity increase the effect of intrinsic noise fluctuations? It was unclear if this co-operative interaction was treated correctly in their reduced model.**

*Some of the low performance can be attributed to variable expression in different slots. For instance, we observe lower expression in the last slot, probably due to the fact that there are two remaining recombination sites left after excision. We have shown recently that these sites can block transcription.*

*Reducing the number of dependent variables for the simplified model means that we no longer have a mechanistic representation of cooperative monomer binding relating to the Cre and Flp recombinases, as already stated in the main text:*

*“The structure of our simplified model network does not retain the mechanistic properties of cooperative monomer binding”*

*The simplified model also loses a mechanistic representation of the two-step Cre-mediated reaction from Z_00_ to Z_10_ which we overcame by setting a requirement for twice as many Cre monomers for that reaction in the Gillespie algorithm simulations. We have added the following comment to the manuscript to clarify this point:*

*“The simplified model also does not retain the two-step Cre-mediated reaction that facilitates the Z_00_ to Z_10_ state transition, however, to rectify this, the number of Cre monomers required to elicit the transition is double the number required for all other transitions within the Gillespie algorithm simulations.”*

**Minor comments**

**I think it will be helpful to the readers if some parts of the text are revised to explain some of the terms used frequently. For example, authors discuss measurements and simulation of in “circuits” using BLADE pattern. Perhaps, introducing the concept of circuits composed of the BLADE platform will be helpful to a non-expert reader.**

*We agree with the reviewer and have added the following comment to page 2 to clarify the point:*

*“The BLADE platform can be used to construct circuits describing any of the 256 (4^4^) possible permutations of four states (states can be repeated). For example the AND (FALSE FALSE FALSE TRUE), OR (FALSE TRUE TRUE TRUE), XOR (FALSE TRUE TRUE FALSE) and NOT (TRUE FALSE) gates that form the basis of logic operations.”*
