# Supplementary material for: A mechanistic model of the BLADE platform predicts performance characteristics of 256 different synthetic DNA recombination circuits
Source: PLoS Comput Biol. 2020 Dec 18;16(12):e1007849. doi: 10.1371/journal.pcbi.1007849 (PMC7781486; doi:10.1371/journal.pcbi.1007849)
Supplement: Attachment — Submitted filename: Reviewer_responses.docx [file pcbi.1007849.s009.docx]

Comments by reviewers are in **bold face**, our response is in *italics*.

**Reviewers responses to questions**

**Comments to the Authors:**

**Reviewer #2**

**The authors have addressed my concerns with their modeling effort. They have both updated their paper, as well as provided matlab code and an SBML model on github. My only last minor concern is it would be good to add a README file on the github repository to explain the files and how to run the analysis to reproduce their results.**

*A README file has now been added to the github repository.*

**Reviewer #3:**

**The authors have addressed most of my comments. In some cases, the text (main text or SI) does not reflect explanations provided in the response letter. For example, for Fig. S4, the readers might benefit from knowing how the authors use Fig. S4 to interpret "robustness" in their parameter choice. The authors state in the response letter (comment #1),

"The histogram bin containing the optimal parameter value is also added, and it can be seen that the bins match up for almost all parameters. This shows that distinct optimisation runs provide similar results, and hence it is likely that the parameterisation we have identified is one of only a small number of similar parameterisations that provides the model outputs reported here. "

Perhaps a brief sentence in the main text or the figure legend of Fig. S4 would help.**

*The following comment has been added to the caption of Figure S4:*

*“the bins match up for almost all parameters. Distinct optimisation runs provide similar results, and hence it is likely that the parameterisation we have identified is one of only a small number of similar parameterisations that provides the model outputs reported here.”* **I think the authors also do not include any potential mechanistic explanation (comment #3) in the main text as they provide in the response,

"Some of the low performance can be attributed to variable expression in different slots. For instance, we observe lower expression in the last slot, probably due to the fact that there are two remaining recombination sites left after excision. We have shown recently that these sites can block transcription."

These mechanistic explanations might be beyond the scope of the work as the authors mention in the letter, therefore, I leave it to the authors if they would like to include this explanation in the text.**

*The following comment has been added to the main text:*

“*Furthermore, we believe some of the low performance can be attributed to variable expression in different address positions. For instance, we observe lower expression in the last position (Z_11_), probably due to the fact that there are two remaining recombination sites left after excision. We have shown recently that these sites can block transcription.”*
